# ARE FEDERAL GUIDELINES HELPING TO IMPROVE PAIN ASSESSMENT, DIAGNOSIS, AND MANAGEMENT IN NURSING HOMES

**DOI:** 10.1093/geroni/igae098.1808

**Published:** 2024-12-31

**Authors:** Sorah Levy

**Affiliations:** University of Maryland School of Nursing, University of Maryland School of Nursing, Maryland, United States

## Abstract

The State Operations Manual for Long Term Care, specifically F-Tag F697, requires that nursing homes must ensure that pain management is provided to residents. The management should be consistent with professional standards of practice, the person-centered care plan, and the residents’ goals and preferences. Although these regulations are required there are many pain related issues not well addressed in the guidance such as recognition of the challenges to pain assessment and management, ways to overcome challenges, how the community incorporates assessment, diagnosis and management of pain using professional standards of practice, the most current standards of practice for long-term care residents, the best ways to manage different types of pain (e.g., neuropathic, chronic), and how to make decisions about changing, adding or stopping an analgesic among others. Further, there are no processes by which the communities or regulatory agencies evaluate if there is a person-centered care plan and if the plan matches the resident’s pain goals. In our pilot work implementing the Pain Management CPG we noted that the majority of the care plans related to pain were generic and not personalized and we were able to help staff make pain care plans more personalized care. The purpose of this paper is to address the mismatch between the requirements and measures within the Minimum Data Set around pain assessment, diagnosis and management and issues and concerns with this approach. We will describe how the Pain Management CPG can help guide communities to overcome these issues and adhere to F-Tag F697.

